# Effects of Food Characteristics on Masticatory and Swallowing Functions in Healthy Young Adults

**DOI:** 10.1111/jtxs.70078

**Published:** 2026-04-08

**Authors:** Rie Koide, Toru Ogawa, Hiroyasu Kanetaka, Taichi Narihara, Hideya Komine, Ryo Tagaino, Ryuji Shigemitsu, Kenta Shobara, Hiroki Hihara, Nobuhiro Yoda

**Affiliations:** ^1^ Division of Advanced Prosthetic Dentistry, Graduate School of Dentistry Tohoku University Sendai Miyagi Japan; ^2^ Division of Comprehensive Dentistry Tohoku University Hospital Sendai Miyagi Japan; ^3^ Division of Orthodontics and Dentofacial Orthopedics, Graduate School of Dentistry Tohoku University Sendai Miyagi Japan; ^4^ Division of Molecular and Regenerative Prosthodontics, Graduate School of Dentistry Tohoku University Sendai Miyagi Japan

**Keywords:** acidity, electromyography, food properties, masticatory function, sensory evaluation, swallowing function

## Abstract

Beyond oral function, the physicochemical properties of food, such as acidity and hardness, significantly influence nutritional intake. Clarifying the relationship between subjective and objective metrics for assessing oral function can help establish simple methods for selecting food with safe and suitable properties tailored to individual oral functions. To objectively and subjectively evaluate the chewing and swallowing processes of gummies with varying hardness and acidity in healthy young adults and to examine relationships between subjective and objective evaluation metrics. Ten healthy young adult men (mean age: 29.8 ± 3.6 years) with no history of oral hypofunction or dysphagia were included. Participants chewed and swallowed four types of gummies (hard with acid, hard without acid, soft with acid, and soft without acid) at their own pace. The subjective evaluation included sensory assessment of chewing and swallowing. Objective evaluations included electromyographic analysis of masticatory and swallowing‐related muscles and analysis of the food properties of gummies that were spat out just before swallowing. Correlations between subjective and objective evaluation metrics were assessed using Peason's correlation analysis. The physical properties and acidity of food significantly affected muscle activity and sensory evaluations of masticatory and swallowing functions. Hard gummies, particularly those without acidity, showed significantly greater masseter and temporalis muscle activity, a higher number of chewing cycles, and longer chewing time than soft gummies. In contrast, no significant differences were observed in swallowing‐related muscle activity or swallowing duration among the gummy types. Furthermore, strong correlations were observed between subjective evaluations and mastication‐related objective parameters, whereas correlations with swallowing‐related metrics were limited. Differences in food hardness caused by the presence or absence of acidity affected the masticatory and swallowing functions of healthy young adults. Furthermore, we identified a relationship between subjective and objective evaluations. Our findings provide fundamental data for investigating the effects of food physical properties on sensory evaluation, mastication, and swallowing functions.

## Graphical abstract

This study assessed chewing and swallowing in healthy young adults using gummies differing in hardness and acidity through one subjective and two objective evaluations. Findings showed that both hardness and acidity influenced masticatory and swallowing functions and revealed relationships among sensory, muscular, and bolus evaluation parameters.

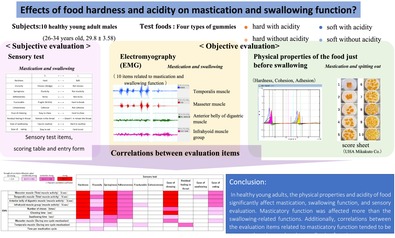

## 1 Introduction

Oral functions, including texture perception, taste, tongue sensation, chewing, bolus formation and transfer, and swallowing, play a crucial role in supporting digestion, immune function, oral hygiene, and overall well‐being. A decline in eating and swallowing functions, including oral function, is closely linked to poor health outcomes such as frailty and malnutrition (Moriya and Miura 2014; Sura et al. 2012). Food characteristics also play major roles in nutritional intake (Pereira et al. 2006; McClements 2024). Therefore, for older adults with weakened oral functions, the optimization of food characteristics with an individual's chewing and swallowing functions is essential for safe oral intake.

Oral health status, including the number of remaining teeth and the use of prostheses, is known to significantly influence masticatory efficiency. However, in the present study, participants were carefully screened, and individuals with oral hypofunction or masticatory impairments were excluded. As a result, no participants met the diagnostic criteria for oral hypofunction, and all participants demonstrated normal masticatory function. This study therefore focused on examining the effects of food characteristics on mastication and swallowing under well‐controlled oral health conditions.

The International Dysphagia Diet Standardisation Initiative (IDDSI) is widely adopted for classifying food texture and consistency in clinical dysphagia management; however, it is primarily intended for safety and practicality rather than for fine‐grained experimental control of food properties. Thus, to address the mechanistic research aims of this study, IDDSI classification was not used, and food samples with systematically manipulated physical properties were prepared.

Food characteristics include nutritional components and physical, sensory, chemical, and biological properties. Among these characteristics, the factors influencing mastication and swallowing primarily include nutritional components and physical properties, including hardness, adhesiveness, and cohesiveness. These physical properties can be quantitatively evaluated using mechanical testing devices to understand how food behaves during oral processing and bolus. Studies have investigated the effects of hardness on chewing function, such as an increase in masticatory muscle activity (Horio and Kawamura 1989; van der Bilt 2011), number of chews, and chewing time (Komino and Shiga 2017).

Chemical characteristics of food, including acidity, capsaicin, and carbonated water, also promote or induce swallowing (Kajii et al. 2002; Sasaki et al. 1997; Krival and Bates 2012; Logemann et al. 1995), with acidity reported to increase saliva volume during mastication (Khramova and Popov 2022). However, it has been reported that few studies have compared samples with different physical properties and compositions within the same food group (Chen 2009). Therefore, it is important to examine the potential influences of both physical properties and taste on mastication and swallowing. Acid reportedly can increase saliva secretion and stimulate the swallowing reflex; however, objective evaluations of these effects are limited, potentially because acid affects the food texture, which can confound the results. Furthermore, how the physical properties of food change during mastication, including characteristics of the food bolus immediately before swallowing, can significantly impact the efficiency of the masticatory and swallowing functions (Ishihara et al. 2011). Thus, understanding these changes is essential. However, the underlying mechanisms underlying oral processing during eating have not yet been fully elucidated (Raheem et al. 2021). In light of this, examining the physical properties of the bolus immediately before swallowing is necessary.

Generally, masticatory and swallowing functions can be evaluated using subjective and objective approaches. Subjective evaluation involves sensory assessments such as texture and ease of eating and swallowing (Szczesniak 2002; Özdoğan et al. 2021). However, food properties are not always consistent with sensory evaluation results (Nishinari et al. 2019). Objective evaluation methods, such as electromyography (EMG) and videofluorography (VF) (Saito et al. 2024; Yokohama et al. 2024), can evaluate mastication and swallowing function and measure the physical properties of food (Shiozawa and Kohyama 2011; Maeda et al. 2020). While recent advances in equipment have simplified these techniques, the methods remain complicated and require specialized knowledge. Furthermore, the relationship between subjective and objective metrics has rarely been investigated, even among healthy adults. Clarifying this relationship can help establish simpler and more accessible methods for selecting safe and suitable food tailored to individual oral functions.

In this study, we collaborated with food manufacturers to create gummies which texture remained unchanged despite the addition of acid. We hypothesized that differences in food hardness and acidity affect chewing and swallowing functions and that a relationship exists between subjective and objective evaluations of chewing and swallowing. Accordingly, we aimed to perform one subjective and two objective evaluations of chewing and swallowing using test gummies with varying hardness and acidity in healthy young adults and clarify the relationships between these evaluation parameters.

## 2 Materials and Methods

### 2.1 Test Foods

Gummies were used as test foods. Four types of gummies with different degrees of hardness and acidity (hard with acidity [H_A], hard without acidity [H_NA], soft with acidity [S_A], and soft without acidity [S_NA]) were used. The acidity was increased by adding citric acid, which also contributed to a pleasant taste. All four gummies were grape flavored and were specially ordered and produced by a food manufacturer (Lotte Co. Ltd., Japan). The size of the gummies (1.5 × 1.5 × 1.5 cm) was the same. Before the experiment, the hardness, adhesiveness, and cohesiveness of the gummies were measured using a creep meter (RE2‐33005B; Yamaden Co. Ltd., Japan) (Shiozawa et al. 2013).

### 2.2 Experimental Design

This study was conducted after obtaining ethical approval for human participation from the Research Ethics Committee of the Graduate School of Dentistry, Tohoku University (Application No. 33017, 8/18/2023). Participants provided written informed consent before the experiment.

This experimental study included 10 healthy young men (26–34 years old, 29.8 ± 3.6) who were recruited. This sampling was by convenience, and participants were recruited from university students and staff through poster advertisement. Based on preliminary experiments indicating differences in muscle activity associated with food texture, the required sample size was calculated to be eight participants to detect a significant difference between two independent groups using a two‐tailed test, with a significance level of 5% and a statistical power of 95% (G*Power version 3.1.9.7). To account for potential dropouts, the sample size was set at 10 participants in the present study.

**FIGURE 1:**
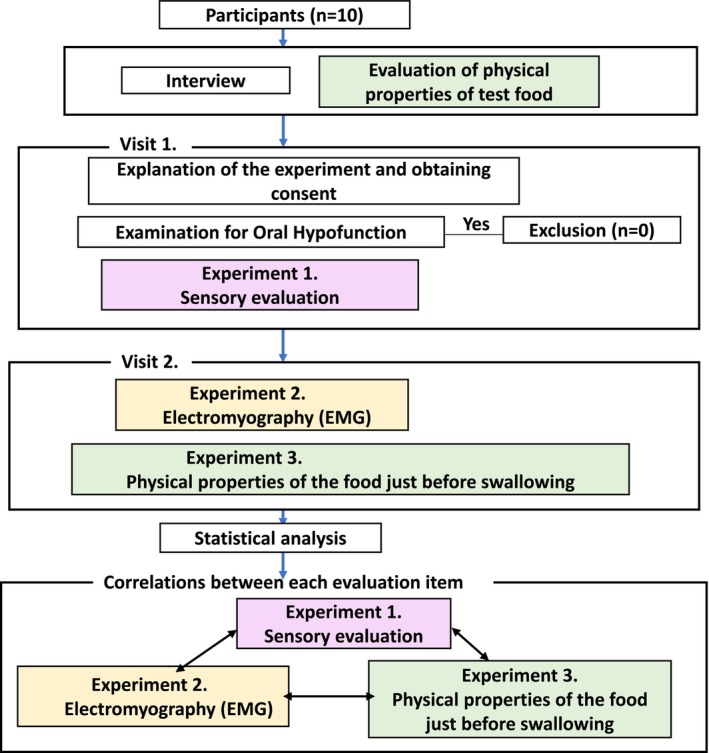
Schema of this study.

Those with oral hypofunction and those with dysphagia were excluded. The absence of dysphagia was confirmed through a dentist‐conducted clinical interview and the Eating Assessment Tool‐10 (EAT‐10), which was completed by participants as a self‐administered questionnaire. Participants were required to have no history of chronic diseases, neurological disorders, or neurodegenerative diseases, and not to be taking any medications. Seven oral signs or symptoms, including oral uncleanness, oral dryness, the decline in occlusal force, the decline in motor function of the tongue and lips, tongue pressure decline, chewing function decline, and swallowing function decline, were examined for oral hypofunction, as defined by the Japanese Society of Geriatric Dentistry (Minakuchi et al. 2018) to ensure the absence of abnormalities in the maxillo–oral region. Oral hypofunction was determined when three of the seven items were applicable (Figure 1 and Table 1). The researchers were trained under the supervision of a licensed dentist based on established protocols, and calibration procedures were performed with guidance from the equipment manufacturer to ensure measurement reliability. Details of the experimental procedure on the day of measurement are provided in the Supporting Information.

**TABLE 1:** Oral hypofunction screening of the study participants.

Clinical signs	Measurements	Average ± SD
Poor oral hygiene	The total number of microorganisms (CFU/mL) is 10^6.5^ or more.	20.0 ± 8.7
Oral dryness	The measured value obtained by a recommended moisture checker is less than 27.0.	27.6 ± 2.0
Reduced occlusal force	The occlusal force is less than 200 N.	951.7 ± 477.1
	Remaining teeth are fewer than 20	28.6 ± 1.3
Decreased tongue‐lip motor function	The number of any counts of /pa/, /ta/ or /ka/ produced per second is less than 6.	/pa/ : 7.1 ± 0.4 /ta/ : 7.6 ± 0.4 /ka/ : 6.9 ± 0.6
Decreased tongue pressure	The maximum tongue pressure is less than 30 kPa.	42.9 ± 5.5
Decreased masticatory function	The glucose concentration obtained by chewing gelatin gummies is less than 100 mg/dL.	245.0 ± 42.0
Deterioration of swallowing function	The total score of EAT‐10 is 3 or higher.	0.0 ± 0.0

### 2.3 Subjective Evaluation

During the experiment, each participant sat upright on a dental chair such that the Frankfurt plane was parallel to the ground (Saito et al. 2024). The gummies were placed on paper plates one at a time in front of the participants. Participants initiated chewing and swallowing at their own time. They were asked to drink pure water before consuming each gummy to eliminate the taste of the previous gummy from their oral cavity.

#### 2.3.1 Sensory Evaluation (Experiment 1)

**FIGURE 2:**
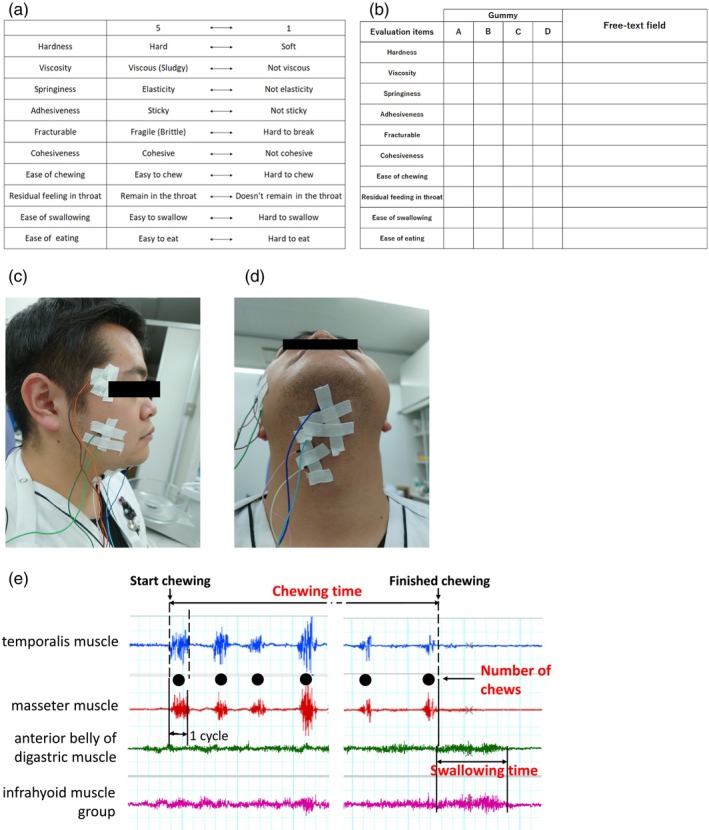
(a) Sensory evaluation items and 5‐point scoring scale. (b) Sensory evaluation form used for participant assessment. (c) Locations of surface EMG electrode placement on the masseter and temporalis muscles. (d) Locations of surface EMG electrode placement on the anterior belly of the digastric muscle and the infrahyoid muscles. (e) EMG analysis parameters and their definitions.

A sensory evaluation was conducted using a scoring method to quantify how the participants felt when they chewed and swallowed each gummy type. The evaluation items were converted from ISO (11,036:2020 Sensory analysis—Methodology—Texture profile) to Japanese. Items related to the ease of swallowing and eating were also included. Ten items on a five‐point scale were used, comprising hardness (5: hard, 1: soft), viscosity (5: viscous (sludgy), 1: not viscous), springiness (5: elasticity, 1: Not elasticity), adhesiveness (5: sticky, 1: not sticky), fracturability (5: fragile (brittle), 1: hard to break), cohesiveness (5: cohesive, 1: not cohesive), ease of chewing (5: easy to chew, 1: hard to chew), residual feeling in the throat (5: remain in the throat, 1: doesn't remain in the throat), ease of swallowing (5: easy to swallow, 1: hard to swallow), and ease of eating (5: easy to eat, 1: hard to eat) (Figure 2a,b). A score of 5 was assigned to the strongest feeling for each item, and a score of 1 was assigned to the weakest feeling.

### 2.4 Objective Evaluation

Participants were instructed to start chewing one gummy at their own pace, chew as they normally would, and to swallow at their self‐determined timing as they normally would in daily eating. They were also instructed to press a button on the computer immediately before chewing initiation and immediately after swallowing to check the timing of chewing initiation and swallowing. Each participant chewed and swallowed each gummy type three times, and the average of the three repetitions was used as the representative for each task. To account for fatigue during the experiment, the order of the gummies was randomized using a random number table.

#### 2.4.1 EMG (Experiment 2)

Surface EMG (sEMG) was used to record activity from the masseter and temporalis muscles, which are involved in mastication, and the anterior belly of the digastric muscle, which is one of the suprahyoid muscles and infrahyoid muscle groups, which are swallowing‐related muscles.

For each muscle, silver/silver chloride surface electrodes (10‐mm diameter, bioelectrode; Nihon Kohden Co. Ltd., Japan) were applied to the skin at two locations, aligned parallel to each muscle bundle. The action potentials of each muscle were bipolarly derived. Each surface electrode was fixed with an adhesive tape (Medipore, 3M Japan Limited, Japan) after cleaning the application site with a skin pretreatment agent (Skinpure; Nihon Kohden Co. Ltd., Japan) and alcohol wipes (Shotmen, White Cross, Japan). A surface electrode with EMG paste (Elefix V, Nihon Kohden Co. Ltd., Japan) was applied, and a ground electrode was attached to the posterior neck (Figure 2c,d) (Long et al. 2018).

Before commencing the experiment, the maximal voluntary muscle activities of the masseter, temporalis, anterior belly of the digastric muscle, and infrahyoid muscle groups were recorded and used to standardize the electromyographic data (Saito et al. 2024). For sEMG recordings of each muscle, a biological amplifier (MEG‐6116 multichannel amplifier; Nihon Kohden Co. Ltd., Japan) was used to filter (5 Hz to 1 kHz) and amplify the signals. After A/D conversion using an A/D converter (Power Lab 16/30, AD Instruments, Japan) with a range of 10 V and a sampling rate of 2 kHz, the data were stored on a personal computer.

#### 2.4.2 Data Analysis

Muscle activity was analyzed using analysis software (Lab Chart8; AD Instruments, Japan), and the outcomes were sEMG bursts of the masseter, temporalis, and anterior belly of the digastric muscles, as well as the infrahyoid muscle group during chewing and swallowing. The parameters were used in the normalized sEMG activity (integrated EMG normalized by maximum sEMG values) and during the sEMG burst of each muscle, using the onset and offset of sEMG activity with swallowing. The duration of the sEMG burst was defined as the time from when the amplitude was > +2 standard deviations (SD) above the resting amplitude to when it was < +2 SD below the resting amplitude. The onset of activity in the anterior belly of the digastric muscle was defined as the onset of swallowing. Swallowing was considered to have ended when the amplitude of the anterior belly of the digastric muscle was < +1 SD of the resting amplitude (Figure 2e). The time from the start of the anterior belly of the digastric muscle to the end of swallowing was defined as the total swallowing duration. Ten items were used for analysis. Specifically, the data included the total muscle activity of the masseter and temporalis muscles during mastication, number of chews, chewing time, muscle activity of the anterior belly of the digastric and infrahyoid muscle groups during swallowing, muscle activity per cycle of mastication of the masseter and temporalis muscles, and time per mastication cycle. The amount of muscle activity per mastication cycle was calculated as the total muscle activity divided by the number of chews, whereas the total muscle activity divided by the chewing time was the time per mastication cycle.

#### 2.4.3 Physical Properties of Food Just Before Swallowing (Experiment 3)

The physical properties of the test gummies, including hardness, adhesiveness, cohesiveness, and size, were evaluated just before swallowing. A creep meter (RE2‐33005B: Yamaden Co. Ltd., Japan) was used to measure hardness, adhesiveness, and cohesiveness following the “Food for People with Dysphagia” standards set by the Ministry of Health, Labour and Welfare, Japan (Maeda et al. 2020; Iguchi et al. 2015). Adhesiveness is a measure of stickiness, whereas cohesiveness is a measure of cohesion. After receiving the gummy, the participants began chewing it at their own time. They chewed the gummies an arbitrary number of times, held 10 mL of water in their oral cavity just before swallowing the gummies, and spat them into a paper cup with gauze. They were also instructed to chew and swallow as they always do when eating. However, no detailed instructions were given, such as to chew thoroughly before swallowing. To further investigate the size of the gummies, the ones that were spit out were spread apart on a gauze using a pair of tweezers and photographed using a camera (Tough TG‐6; Olympus Corp., Japan). The gummies were transferred to a Petri dish to measure their physical properties. The Petri dish was made of stainless steel (40 mm in diameter and 15 mm in height), and the plunger was made of acrylic resin with a diameter of 20 mm, clearance of 5 mm, and compression speed of 10 mm/s.

#### 2.4.4 Data Analysis

The hardness, adhesiveness, and cohesiveness were calculated from the texture curves obtained (Texture Analysis ver. 2.0, Yamaden Corporation, Japan) following their measurements (Kochi et al. 2021). The size of the gummies was evaluated through visual scoring using images captured with a camera on a previously established and standardized 10‐point scale (score sheet, UHA Mikakuto Co. Ltd., Japan) (Nokubi et al. 2010, 2013; Gonçalves et al. 2021).

### 2.5 Statistical Analysis

Statistical analysis of the results of each experiment was performed using repeated‐measures one‐way analysis of variance and multiple comparison Bonferroni tests (SPSS Statistics v29.0; IBM Corp.). Normality was assessed using the Shapiro–Wilk test. As the data were normally distributed, parametric statistical analyses were performed. The significance level was set at 5%. Additionally, Pearson's correlation was used to evaluate the correlation between each evaluation item, including sensory and EMG evaluation, sensory evaluation and physical properties of the tested food immediately before swallowing, and EMG evaluation and physical properties of the tested food immediately before swallowing (Microsoft Excel version 16.0; Microsoft Corporation, Redmond, WA, USA).

## 3 Results

The participants were 10 healthy young adult men (mean age: 29.8 ± 3.6 years). All participants were confirmed not to meet the criteria for oral hypofunction in the screening assessment and had no relevant medical conditions, no regular medication use, or special dietary restrictions.

### 3.1 Subjective Evaluation

#### 3.1.1 Sensory Evaluation (Experiment 1)

An average of 10 results was obtained for each of the four gummies (Table 2a). The hardness and springiness of hard gummies significantly differed (between H_A and S_A). Similarly, viscosity, ease of swallowing, and ease of eating significantly differed for hard gummies (H_A and H_NA) with and without acidity. For the other items, no significant differences were observed between any of the gummies or between gummies with two different gummy elements (between H_A and S_NA and between H_NA and S_A). The results showed that participants could perceive hardness and springiness as textures, and some feelings were perceived differently in the absence or presence of acidity.

**TABLE 2:** (a) Results of sensory evaluation. (b) Results of EMG. (c) Results of physical properties of food just before swallowing.

*(a) Sensory evaluation*	H_A means ± SD	H_NA means ± SD	S_A means ± SD	S_NA means ± SD	*p*
Hardness	3.4 ± 0.70	4.2 ± 0.63	1.5 ± 0.71	1.9 ± 0.74	*a* > *c* :0.003**, *b* > *c* :0.004*,
Viscosity	2.7 ± 0.67	1.8 ± 0.79	3.0 ± 1.56	2.9 ± 1.20	*a* > *b* :0.023*
Springiness	3.6 ± 0.97	4.2 ± 0.92	1.9 ± 0.88	2.7 ± 1.25	*a* < *c* :0.012*, *b* > *c* :0.011*
Adhesiveness	2.6 ± 0.84	1.7 ± 0.67	3.4 ± 1.26	2.8 ± 1.03	*b* < *c* :0.035*
Fracturable	3.1 ± 0.74	2.9 ± 1.10	3.6 ± 1.26	3.4 ± 1.17	n.s.
Cohesiveness	3.0 ± 1.33	2.2 ± 0.92	3.0 ± 1.25	3.0 ± 1.41	n.s.
Ease of chewing	3.8 ± 0.63	2.7 ± 1.06	4.4 ± 0.70	4.1 ± 0.74	*b* < *c* :0.012*
Residual feeling in throat	2.2 ± 1.03	2.4 ± 1.07	2.7 ± 1.70	2.5 ± 1.18	n.s.
Ease of swallowing	4.2 ± 0.79	3.2 ± 1.23	3.9 ± 1.10	3.6 ± 0.84	*a* > *b* :0.049*
Ease of eating	3.9 ± 0.57	2.6 ± 0.70	4.1 ± 0.88	3.8 ± 1.03	*a* > *b* :0.001**, *b* < *c* :0.018*
*(b) EMG*					
Masseter muscle (total muscle activity) (V s)	27.98 ± 14.32	40.28 ± 20.69	14.89 ± 7.30	22.46 ± 12.59	*a* < *b* :0.009**, *a* > *c* :0.004**, *a* > *d* :0.023*, *b* > *c* :0.003**, *b* > *d* :0.002**, *c* < *d* :0.025*
Temporalis muscle (total muscle activity) (V s)	33.28 ± 18.91	46.63 ± 22.63	18.05 ± 9.45	25.81 ± 11.90	*a* < *b* :0.002**, *a* > *c* :0.014*, *b* > *c* :0.002**, *b* > *d* :0.003**, *c* < *d* :0.003**
Number of chews (times)	50.00 ± 16.07	64.90 ± 20.64	32.50 ± 9.67	45.00 ± 12.59	*a* < *b* :0.006**, *a* > *c* :0.002**, *b* > *c* :0.001**, *b* > *d* :0.004**, *c* < *d* :0.001**
Chewing time (s)	29.19 ± 8.20	39.69 ± 12.46	18.15 ± 5.19	25.30 ± 6.23	*a* < *b* :0.008**, *a* > *c* :0.001**, *b* > *c* :0.001**, *b* > *d* :0.003**, *c* < *d* : 0.001**
Masseter muscle (during one‐cycle mastication) (V s/times)	0.59 ± 0.29	0.67 ± 0.40	0.43 ± 0.17	0.46 ± 0.17	*c* < *d* :0.011*
Temporalis muscle (during one‐cycle mastication) (V s/times)	0.70 ± 0.35	0.77 ± 0.34	0.57 ± 0.29	0.61 ± 0.31	n.s.
Time per mastication cycle (s/times)	0.59 ± 0.05	0.62 ± 0.08	0.56 ± 0.04	0.57 ± 0.05	*b* > *d* :0.004**
Anterior belly of digastric muscle (muscle activity) (V s)	1.37 ± 0.68	1.51 ± 0.77	1.26 ± 0.86	1.31 ± 0.66	n.s.
Infrahyoid muscle group (muscle activity) (V s)	1.51 ± 0.73	1.56 ± 0.78	1.32 ± 0.84	1.33 ± 0.75	n.s.
Swallowing time (s)	0.76 ± 0.23	0.80 ± 0.22	0.74 ± 0.25	0.77 ± 0.21	n.s.
*(c) Physical properties of food just before swallowing*					
Hardness (N/m^2^)	2.2 × 10^3^ ± 1.2 × 10^3^	1.4 × 10^3^ ± 6.8 × 10^2^	2.0 × 10^3^ ± 6.9 × 10^2^	1.3 × 10^3^ ± 6.9 × 10^2^	*a* > *b* :0.049*, *a* > *c* :0.005**, *c* > *d* :0.023*
Cohesiveness	0.62 ± 0.10	0.61 ± 0.10	0.73 ± 0.07	0.67 ± 0.04	*b* < *c* :0.017*
Adhesiveness (J/m^3^)	84.16 ± 50.95	54.24 ± 24.39	165.12 ± 40.27	91.43 ± 31.43	*a* < *c* :0.012*, *b* > *c* :0.011*
Size	8.1 ± 0.89	7.6 ± 1.06	8.0 ± 0.63	8.0 ± 0.93	*a* < *c* :0.001**, *b* < *c* :0.001**, *b* < *d* :0.001**, *c* > *d* :0.001**

*Note:* * *p* < 0.05 ** *p* < 0.01.

### 3.2 Objective Evaluations

#### 3.2.1 EMG (Experiment 2)

Rhythmic activities of the masseter and temporalis were observed when the gummy was chewed. Following these activities, distinct activities in the swallowing‐related muscles were observed during swallowing. The average value for each evaluation item was calculated for the four gummy types of the 10 participants. The total muscle activity of the masseter and temporalis muscles during chewing, the number of chews, and the chewing time were significantly greater for hard gummies than for soft gummies, as well as gummies without acidity than for those with acidity. Among all gummies, H_NA exhibited the greatest value for these metrics (Table 2b). By contrast, no significant differences were observed in the muscle activity of the anterior belly of the digastric and infrahyoid muscle groups during swallowing or in swallowing time between the gummy types. Furthermore, during mastication, the muscle activity and time of the masseter and temporalis muscles per cycle of mastication were analyzed. The activity of the masseter muscle per mastication cycle was significantly higher with S_NA than with S_A for soft gummies (between S_A and S_NA), indicating that the load on the masseter muscle per mastication cycle was greater in the absence of acidic gummies. H_NA gummies required significantly more time per cycle of mastication (between H_NA and S_NA), indicating that harder gummies without acidity required more time.

#### 3.2.2 Physical Properties of the Food Just Before Swallowing (Experiment 3)

The spit‐out gummy morsels were easily separated into individual grains using tweezers. The average values of each physical property of the 10 participants were obtained for each gummy type (hardness, adhesiveness, cohesiveness, and size) and compared with the values among the groups (Table 2c). In hard (H_A and H_NA) and soft (S_A and S_NA) gummies that were spit out just before swallowing, those with acidity were significantly greater than those without. Adhesiveness and cohesiveness remained almost unchanged immediately before swallowing. Furthermore, hardness and adhesiveness decreased significantly during chewing. Size did not significantly differ among the four gummy types, indicating they were swallowed at approximately the same size.

### 3.3 Correlations Between Subjective and Objective Evaluations

**FIGURE 3:**
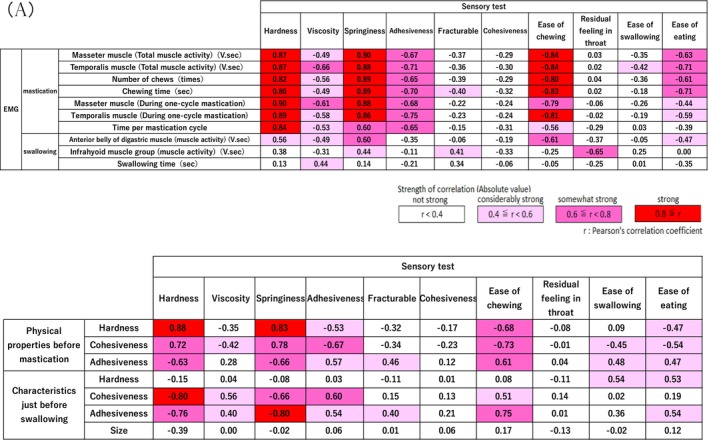
Correlations between each evaluation item. (a) EMG and sensory evaluation. (b) Physical properties and sensory evaluation.

The correlation between sensory evaluation and EMG analysis items was strong, especially for mastication‐related EMG analysis parameters. Among the sensory evaluation items, fracturability, cohesiveness, and ease of swallowing had weaker correlations with the EMG analysis parameters than with the other parameters (Figure 3a). The hardness and springiness of the sensory evaluation were strongly correlated with the three physical properties of the gummies before chewing and the physical properties of the gummies that were spat out just before swallowing (cohesiveness and adhesiveness) (Figure 3b). However, the sensory evaluation was weakly correlated with the physical properties (hardness and size) of the gummy that was spit out just before swallowing.

## 4 Discussion

This study aimed to clarify the effects of food hardness and acidity on masticatory and swallowing functions in healthy young adults and to examine the relationships between subjective and objective evaluations. The results demonstrated that hardness and acidity significantly affected masticatory behavior and sensory perception, with strong correlations between subjective evaluations and mastication‐related objective evaluations, whereas swallowing‐related parameters showed no significant differences.

### 4.1 Sensory Evaluation

Participants' perceptions of chewing and swallowing test foods were assessed with a 10‐point scale, including texture, ease of swallowing, and ease of eating, to compare effects on participants' subjective evaluations. Hardness and springiness—physical oral sensory stimuli characterizing food—differed among gummies of varying hardness and are thus factors enabling differentiation. Reportedly, hardness and springiness significantly affect perceived food texture, consistent with our findings (Smirnov et al. 2024). Interestingly, significant differences in viscosity, ease of swallowing, and ease of eating were observed between hard gummies with and without acidity, suggesting that texture and acidity affect chewing and swallowing. More viscous food remains longer in the mouth (Kohyama 2015), and acids promote saliva secretion (Bozorgi et al. 2020); thus, acidic gummies are more easily swallowed. Acidic hard gummies were easier to swallow and eat, suggesting that chewing and swallowing were less challenging, consistent with previous reports showing that acidity increases saliva volume and induces the swallowing reflex (Logemann et al. 1995; Palmer et al. 2005; Inoue 2021).

### 4.2 EMG

Herein, we focused on masticatory (masseter and temporalis muscles) and swallowing‐related (the anterior belly of digastric and infrahyoid muscle groups) muscles, commonly investigated previously (Saito et al. 2024). The total activity of masseter and temporalis muscles, number of chews, and chewing time were significantly greater for hard gummies than for soft gummies, consistent with previous reports (Horio and Kawamura 1989; Tonni et al. 2020). Conversely, swallowing‐related muscle activity during swallowing or swallowing time did not significantly differ among the four gummy types. Thus, muscle activity during swallowing has a minimal relationship with gummy characteristics. For stable swallowing, the masticatory function is likely adapted to gummy characteristics, resulting in the formation of a food bolus suitable for swallowing, consistent with previous studies (Kohyama et al. 2006). Furthermore, the activity of masseter and temporalis muscles, number of chews, and chewing time were significantly higher for gummies without acidity, which likely require more muscle activity and time for chewing, indicating the influence of acidity on masticatory function. This may be related to the result that gummies with acidity were rated higher in terms of ease of eating in subjective evaluations. Masticatory movements exhibit a unique pattern and rhythm in the same individual for a given food (Gibbs et al. 1971); masticatory function is also affected by food characteristics (Shiozawa et al. 2003). However, swallowing‐related EMG parameters herein did not significantly differ between test gummies. Therefore, under the conditions herein, the mastication process was adapted to gummy characteristics, leading to constant and stable swallowing muscle activity.

### 4.3 Physical Properties of Food Just Before Swallowing

Texture analyzers are commonly used to measure the hardness, adhesiveness, and cohesiveness of test foods in Europe; however, a creep meter, also used here, reliably measures these fundamental physical properties (Maeda et al. 2020). Gummy hardness decreased with chewing time because chewing made the gummies finer and reduced their physical strength, indicating a transformation into a state suitable for swallowing. Furthermore, compared to those without acidity, hard and soft gummies with acidity were significantly firmer immediately before swallowing. Adhesion decreased significantly in all gummies immediately before swallowing, partly owing to the increased lubricity of the food bolus caused by the saliva. Saliva enhances the lubrication of the food bolus because more saliva is secreted in response to acidity. Furthermore, salivary secretion is known to be promoted by acids, which may have facilitated the earlier initiation of swallowing, even though the bolus remained relatively firm. This highlights mastication's role in transforming food into a swallowing form, such as a food bolus (Hutchings and Lillford 1988; Nishinari et al. 2024). Herein, gelatin‐based gummies, generally considered highly elastic, were used as the test food. Their cohesiveness did not change due to their high elasticity, even if they were made finer by chewing. The size of the four gummy types did not differ immediately before swallowing (Peyron et al. 2004; Stokes et al. 2013). By contrast, differences were observed in hardness and adhesiveness, suggesting that for gummy‐like food, the size of food pieces triggered the swallowing reflex (van der Bilt et al. 1993; Jalabert‐Malbos et al. 2007).

### 4.4 Correlations Between Evaluation Parameters

We found that sensory evaluations were strongly correlated with EMG analysis, especially those related to mastication. Specifically, perceived hardness, springiness, and adhesiveness were strongly associated with masticatory muscle activity, including that of masseter and temporalis muscles. Nevertheless, sensory evaluations of fragility, cohesiveness, and residual throat sensations showed weaker correlations. Because these items may be influenced by changes in physical properties during chewing and food condition during swallowing (Shiozawa et al. 2003; Peyron et al. 2011), the simple, uniform composition and elastic properties of gummies may have contributed to these results. Furthermore, EMG evaluation of swallowing‐related items tended to have a lower correlation with sensory evaluation. This may be because chewing that was adapted to gummy characteristics helped to maintain a stable swallowing function.

Herein, a strong correlation was observed between sensory evaluation items such as hardness and springiness and the cohesiveness and adhesiveness of a gummy spit out just before swallowing. The physical hardness showed a high correlation with the hardness, springiness, and ease of chewing in the sensory evaluation of the properties of the original gummy but little correlation with the hardness in the sensory evaluation just before swallowing. This finding suggests that hardness changed significantly with chewing. However, different trends in cohesiveness and adhesiveness, which remained highly correlated, were observed immediately before swallowing, suggesting that trends in these groups of physical properties remained unchanged before and after chewing.

Parameters showing strong correlations with the sensory evaluations related to chewing, masticatory muscle activity, and food properties were identified. However, the correlation between swallowing‐related EMG parameters and sensory evaluations was not as high as that of mastication‐related EMG parameters. Nonetheless, throat sensation, a sensory evaluation item, showed a relatively high correlation with swallowing‐related muscle activity, suggesting its potential for assessing swallowing function. These findings indicate that safe food selection tailored to oral function can be achieved using sensory evaluation parameters with strong correlations.

### 4.5 Broader Implications and Scientific Contribution

This study contributes to scientific advancement by clarifying the relationship between subjective sensory perception and objective physiological responses during oral processing under well‐controlled food conditions. The findings indicate that mastication‐related EMG parameters, rather than swallowing‐related measures, more sensitively reflect differences in food hardness and acidity in healthy young adults. This insight refoorts the development of simplified evaluation strategies for oral function and food selection, which may be particularly valuable in translational research linking food design, sensory assessment, and oral physiology.

### 4.6 Limitations

This study has some limitations. Notably, participants were limited to men for three main reasons. First, men generally have more muscle mass than women (Tanimoto et al. 2007), making muscle activity easier to record clearly. Second, women have more confounding factors, such as hormonal balance, that affect muscle activity, necessitating a larger sample size. Third, the movement of the thyroid cartilage and hyoid bone may make it difficult to detect muscle activity in women (Matsuyama et al. 2021). Moreover, the relatively small sample size (*n* = 10) and the inclusion of only healthy young men limit the generalizability of the present findings. Therefore, the results should be interpreted with caution and are primarily applicable to healthy young men populations, and further investigation using more diverse participant groups, including women and older adults, is required to clarify the broader applicability of these results.

In addition, only EMG evaluation was performed in the swallowing evaluation. This was because masticatory and swallowing evaluations were performed simultaneously herein, and there was concern that VF would affect masticatory function. Although the influence of acidity and salivary secretion on oral processing was considered, a limitation of this study is that salivary volume during mastication was not measured. Quantitative assessment of salivary flow often requires saliva collection procedures or the insertion of devices into the oral cavity. When performed concurrently with chewing and swallowing tasks, these procedures may interfere with natural chewing patterns and alter the timing of swallowing, thereby deviating from habitual eating behavior. Therefore, in the present study, salivary flow was not measured simultaneously, and priority was given to evaluating mastication and swallowing under conditions that closely reflect natural oral behavior.

Furthermore, the method of measuring physical properties immediately before swallowing, just before the gummy was spit out, is not a measure of true physical properties, considering all gummies in the mouth were examined after chewing and participants were given 10 mL of water to spit out the gummies. This additional water intake meant that the measured physical properties might not fully represent actual bolus characteristics immediately before swallowing.

Additionally, we did not directly measure the water content of the expectorated bolus, which could reflect the degree of salivary secretion induced by mechanical stimulation (chewing) and chemical stimulation (acidity). Quantifying the water content would provide further insight into the influence of acidity and food texture on bolus formation. Moreover, the test food was limited to gummies, which do not replicate the variety of textures and heterogeneity of common food because the physical properties of gummies are uniform. Finally, the physical properties of the test food, including hardness and acidity, were controlled. Therefore, the cohesiveness and adhesiveness of the test food were not under constant control among the four gummy types.

### 4.7 Future Directions and Implications

A logical subsequent study is to replicate the present protocol in healthy older adults using the same gelatin‐based gummies and experimental procedures, and to directly compare age‐related differences in subjective sensory ratings, mastication‐related EMG parameters, and bolus properties immediately before swallowing. Such an age‐group comparison would clarify whether the mastication‐related EMG measures identified as sensitive in young adults remain informative in older adults. Building on this foundation, future studies may further expand the range of test foods to include diverse textures beyond gelatin‐based gummies. By leveraging sensory evaluation, which showed strong correlations with objective measures in the present study, it may be possible to develop simplified screening tools to support the selection of safe and appropriate foods tailored to individual oral capabilities. Although acidity may influence salivary secretion and oral pH, the potential relationship between acidulant use and dental caries risk was beyond the physiological scope of the present study. Future studies incorporating direct oral pH measurements and clinical dental outcomes are warranted to clarify this issue.

## 5 Conclusion

We discovered that differences in food hardness, with or without acidity, affected masticatory and swallowing functions in healthy young adults. Because the participants were healthy young adults, no significant differences were observed among gummies in muscle activity for swallowing, owing to the appropriate masticatory function for safe swallowing. Furthermore, we observed a relationship between subjective and objective evaluations of chewing and swallowing of the tested gummies. Therefore, mastication‐related EMG parameters may be more useful as objective indicators for assessing food texture and oral processing of food because they show a stronger correlation with the physical properties and sensory evaluation of food than swallowing‐related EMG parameters. The study findings provide fundamental data for investigating effects of physical properties of food on sensory evaluation, mastication function, and swallowing function.

## Author Contributions

**Rie Koide:** conceptualization, methodology, validation, investigation, writing – original draft, funding acquisition. **Toru Ogawa:** conceptualization, methodology, validation, formal analysis, writing – original draft, funding acquisition, project administration. **Hiroyasu Kanetaka:** conceptualization, methodology, validation, resources, writing – review and editing, project administration. **Taichi Narihara:** validation, investigation. **Hideya Komine:** validation, investigation. **Ryo Tagaino:** methodology, validation, writing – review and editing. **Ryuji Shigemitsu:** writing – review and editing. **Kenta Shobara:** methodology. hiroki hihara: methodology. **Nobuhiro Yoda:** writing – review and editing, project administration.

## Funding

This work was supported by the Ministry of Education, Culture, Sports, Science and Technology (MEXT) / Japan Society for the Promotion of Science (JSPS) WISE Program (Doctoral Program for World‐leading Innovative & Smart Education), Tohoku University; the Japan Science and Technology Agency (JST) SPRING program (Support for Pioneering Research Initiated by the Next Generation, JPMJSP2114); and the Japan Society for the Promotion of Science (JSPS) KAKENHI (JP22K10092).

## Disclosure

No artificial intelligence (AI) assisted technologies were used in the research or writing process of this manuscript.

## Ethics Statement

This study was conducted in accordance with the Declaration of Helsinki and was approved by the Research Ethics Committee of the Graduate School of Dentistry, Tohoku University (Application No. 33017; approved on August 18, 2023).

## Consent

Participants provided written informed consent before the experiment.

## Conflicts of Interest

The authors declare no conflicts of interest.

## Supporting information


**Data S1:** jtxs70078‐sup‐0001‐supinfo.xlsx.

## Data Availability

The data that support the findings of this study are available on request from the corresponding author. The data are not publicly available due to privacy or ethical restrictions.

## References

[jtxs70078-bib-0001] Bozorgi, C. , C. Holleufer , and K. Wendin . 2020. “Saliva Secretion and Swallowing‐The Impact of Different Types of Food and Drink on Subsequent Intake.” Nutrients 12, no. 1: 256. 10.3390/nu12010256. 31963804 PMC7019672

[jtxs70078-bib-0002] Chen, J. 2009. “Food Oral Processing—A Review.” Food Hydrocolloids 23, no. 1: 1–25. 10.1016/j.foodhyd.2007.11.013.

[jtxs70078-bib-0003] Gibbs, C. H. , T. Messerman , J. B. Reswick , and H. J. Derda . 1971. “Functional Movements of the Mandible.” Journal of Prosthetic Dentistry 26, no. 6: 604–620. 10.1016/0022-3913(71)90085-0. 5287059

[jtxs70078-bib-0004] Gonçalves, T. M. S. V. , M. Schimmel , A. van der Bilt , et al. 2021. “Consensus on the Terminologies and Methodologies for Masticatory Assessment.” Journal of Oral Rehabilitation 48, no. 6: 745–761. 10.1111/joor.13161. 33638156 PMC8252777

[jtxs70078-bib-0005] Horio, T. , and Y. Kawamura . 1989. “Effects of Texture of Food on Chewing Patterns in the Human Subject.” Journal of Oral Rehabilitation 16, no. 2: 177–183. 10.1111/j.1365-2842.1989.tb01331.x. 2715866

[jtxs70078-bib-0006] Hutchings, J. B. , and P. J. Lillford . 1988. “The Perception of Food Texture—The Philosophy of the Breakdown Path.” Journal of Texture Studies 19, no. 2: 103–115. 10.1111/j.1745-4603.1988.tb00928.x.

[jtxs70078-bib-0007] Iguchi, H. , J. Magara , Y. Nakamura , T. Tsujimura , K. Ito , and M. Inoue . 2015. “Changes in Jaw Muscle Activity and the Physical Properties of Foods With Different Textures During Chewing Behaviors.” Physiology and Behavior 152, no. A: 217–224. 10.1016/j.physbeh.2015.10.004. 26440319

[jtxs70078-bib-0008] Inoue, M. 2021. “Molecular Physiology of Pharyngeal/Laryngeal Sensory Systems Involved in Swallowing Initiation.” Japanese Journal of Rehabilitation Medicine 58, no. 1: 11–18. 10.2490/jjrmc.58.11.

[jtxs70078-bib-0009] Ishihara, S. , M. Nakauma , T. Funami , S. Odake , and K. Nishinari . 2011. “Swallowing Profiles of Food Polysaccharide Gels in Relation to Bolus Rheology.” Food Hydrocolloids 25, no. 5: 1016–1024. 10.1016/j.foodhyd.2010.09.022.

[jtxs70078-bib-0010] Jalabert‐Malbos, M.‐L. , A. Mishellany‐Dutour , A. Woda , and M.‐A. Peyron . 2007. “Particle Size Distribution in the Food Bolus After Mastication of Natural Foods.” Food Quality and Preference 18, no. 5: 803–812. 10.1016/j.foodqual.2007.01.010.

[jtxs70078-bib-0011] Kajii, Y. , T. Shingai , J.‐I. Kitagawa , et al. 2002. “Sour Taste Stimulation Facilitates Reflex Swallowing From the Pharynx and Larynx in the Rat.” Physiology and Behavior 77, no. 2–3: 321–325. 10.1016/s0031-9384(02)00854-5. 12419408

[jtxs70078-bib-0012] Khramova, D. S. , and S. V. Popov . 2022. “A Secret of Salivary Secretions: Multimodal Effect of Saliva in Sensory Perception of Food.” European Journal of Oral Sciences 130, no. 2: e12846. 10.1111/eos.12846. 34935208

[jtxs70078-bib-0013] Kochi, I. , E. Takei , R. Maeda , et al. 2021. “Changes of Bolus Properties and the Triggering of Swallowing in Healthy Humans.” Journal of Oral Rehabilitation 48, no. 5: 592–600. 10.1111/joor.13151. 33481324

[jtxs70078-bib-0014] Kohyama, K. 2015. “Oral Sensing of Food Properties.” Journal of Texture Studies 46, no. 3: 138–151. 10.1111/jtxs.12099.

[jtxs70078-bib-0015] Kohyama, K. , H. Sawadai , M. Nonaka , and M. Nakajoh . 2006. “Texture Evaluation of Rice Cake for People With Mastication Difficulty and Dysphagia by Instrumental Analysis and Human Measurement During Eating.” Japanese Journal of Dysphagia Rehabilitation 10: 115–124.

[jtxs70078-bib-0016] Komino, M. , and H. Shiga . 2017. “Changes in Mandibular Movement During Chewing of Different Hardness Foods.” Odontology 105, no. 4: 418–425. 10.1007/s10266-016-0292-z. 28150182 PMC5639017

[jtxs70078-bib-0017] Krival, K. , and C. Bates . 2012. “Effects of Club Soda and Ginger Brew on Linguapalatal Pressures in Healthy Swallowing.” Dysphagia 27, no. 2: 228–239. 10.1007/s00455-011-9358-9. 21811834

[jtxs70078-bib-0018] Logemann, J. A. , B. R. Pauloski , L. Colangelo , C. Lazarus , M. Fujiu , and P. J. Kahrilas . 1995. “Effects of a Sour Bolus on Oropharyngeal Swallowing Measures in Patients With Neurogenic Dysphagia.” Journal of Speech and Hearing Research 38, no. 3: 556–563. 10.1044/jshr.3803.556. 7674647

[jtxs70078-bib-0019] Long, J. , T. Ogawa , T. Ito , et al. 2018. “Effect of Bite Openings and Mandibular Protrusion on Genioglossus Muscle Activity in Healthy Adults With Oral Appliance.” Odontology 106, no. 1: 90–95. 10.1007/s10266-017-0299-0. 28215005

[jtxs70078-bib-0020] Maeda, R. , E. Takei , K. Ito , J. Magara , T. Tsujimura , and M. Inoue . 2020. “Inter‐Individual Variation of Bolus Properties in Triggering Swallowing During Chewing in Healthy Humans.” Journal of Oral Rehabilitation 47, no. 9: 1161–1170. 10.1111/joor.13044. 32621336

[jtxs70078-bib-0021] Matsuyama, S. , M. Nakauma , T. Funami , K. Hori , and T. Ono . 2021. “Human Physiological Responses During Swallowing of Gel‐Type Foods and Its Correlation With Textural Perception.” Food Hydrocolloids 111: 106353. 10.1016/j.foodhyd.2020.106353.

[jtxs70078-bib-0022] McClements, D. J. 2024. “Designing Healthier and More Sustainable Ultraprocessed Foods.” Comprehensive Reviews in Food Science and Food Safety 23, no. 2: e13331. 10.1111/1541-4337.13331. 38517032

[jtxs70078-bib-0023] Minakuchi, S. , K. Tsuga , K. Ikebe , et al. 2018. “Oral Hypofunction in the Older Population: Position Paper of the Japanese Society of Gerodontology in 2016.” Gerodontology 35, no. 4: 317–324. 10.1111/ger.12347. 29882364

[jtxs70078-bib-0024] Moriya, S. , and H. Miura . 2014. “Oral Health and General Health at the Early Stage of Ageing: A Review of Contemporary Studies.” Japanese Dental Science Review 50, no. 1: 15–20. 10.1016/j.jdsr.2013.10.002.

[jtxs70078-bib-0025] Nishinari, K. , Y. Fang , and A. Rosenthal . 2019. “Human Oral Processing and Texture Profile Analysis Parameters: Bridging the Gap Between the Sensory Evaluation and the Instrumental Measurements.” Journal of Texture Studies 50, no. 5: 369–380. 10.1111/jtxs.12404. 31008516

[jtxs70078-bib-0026] Nishinari, K. , M.‐A. Peyron , N. Yang , et al. 2024. “The Role of Texture in the Palatability and Food Oral Processing.” Food Hydrocolloids 147: 109095. 10.1016/j.foodhyd.2023.109095.

[jtxs70078-bib-0027] Nokubi, T. , F. Nokubi , Y. Yoshimuta , K. Ikebe , T. Ono , and Y. Maeda . 2010. “Measuring Masticatory Performance Using a New Device and β‐Carotene in Test Gummy Jelly.” Journal of Oral Rehabilitation 37, no. 11: 820–826. 10.1111/j.1365-2842.2010.02112.x. 20557437

[jtxs70078-bib-0028] Nokubi, T. , Y. Yoshimuta , F. Nokubi , et al. 2013. “Validity and Reliability of a Visual Scoring Method for Masticatory Ability Using Test Gummy Jelly.” Gerodontology 30, no. 1: 76–82. 10.1111/j.1741-2358.2012.00647.x. 22471409

[jtxs70078-bib-0029] Özdoğan, G. , X. Lin , and D.‐W. Sun . 2021. “Rapid and Noninvasive Sensory Analyses of Food Products by Hyperspectral Imaging: Recent Application Developments.” Trends in Food Science and Technology 111: 151–165. 10.1016/j.tifs.2021.02.044.

[jtxs70078-bib-0030] Palmer, P. M. , T. M. McCulloch , D. Jaffe , and A. T. Neel . 2005. “Effects of a Sour Bolus on the Intramuscular Electromyographic (EMG) Activity of Muscles in the Submental Region.” Dysphagia 20, no. 3: 210–217. 10.1007/s00455-005-0017-x. 16362509

[jtxs70078-bib-0031] Pereira, L. J. , M. B. D. Duarte Gaviao , and A. Van Der Bilt . 2006. “Influence of Oral Characteristics and Food Products on Masticatory Function.” Acta Odontologica Scandinavica 64, no. 4: 193–201. 10.1080/00016350600703459. 16829493

[jtxs70078-bib-0033] Peyron, M. A. , A. Mishellany , and A. Woda . 2004. “Particle Size Distribution of Food Boluses After Mastication of Six Natural Foods.” Journal of Dental Research 83, no. 7: 578–582. 10.1177/154405910408300713. 15218050

[jtxs70078-bib-0032] Peyron, M.‐A. , I. Gierczynski , C. Hartmann , et al. 2011. “Role of Physical Bolus Properties as Sensory Inputs in the Trigger of Swallowing.” PLoS One 6, no. 6: e21167. 10.1371/journal.pone.0021167. 21738616 PMC3124480

[jtxs70078-bib-0034] Raheem, D. , C. Carrascosa , F. Ramos , A. Saraiva , and A. Raposo . 2021. “Texture‐Modified Food for Dysphagic Patients: A Comprehensive Review.” International Journal of Environmental Research and Public Health 18, no. 10: 5125. 10.3390/ijerph18105125. 34066024 PMC8150365

[jtxs70078-bib-0035] Saito, N. , T. Ogawa , N. Shiraishi , et al. 2024. “Difference in the Electromyographic Behavior of the Masticatory and Swallowing Muscles During Cued Versus Spontaneous Swallowing.” Dysphagia 39, no. 3: 398–406. 10.1007/s00455-023-10621-x. 37752277 PMC11127863

[jtxs70078-bib-0036] Sasaki, H. , K. Sekizawa , M. Yanai , H. Arai , M. Yamaya , and T. Ohrui . 1997. “New Strategies for Aspiration Pneumonia.” Internal Medicine (Tokyo, Japan) 36, no. 12: 851–855. 10.2169/internalmedicine.36.851. 9475237

[jtxs70078-bib-0037] Shiozawa, K. , and K. Kohyama . 2011. “Effects of Addition of Water on Masticatory Behavior and the Mechanical Properties of the Food Bolus.” Journal of Oral Biosciences 53, no. 2: 148–157. 10.1016/S1349-0079(11)80018-6.

[jtxs70078-bib-0038] Shiozawa, K. , K. Kohyama , and K. Yanagisawa . 2003. “Relationship Between Physical Properties of a Food Bolus and Initiation of Swallowing.” Japanese Journal of Oral Biology 45, no. 2: 59–63. 10.2330/joralbiosci1965.45.59.

[jtxs70078-bib-0039] Shiozawa, M. , H. Taniguchi , H. Hayashi , et al. 2013. “Differences in Chewing Behavior During Mastication of Foods With Different Textures.” Journal of Texture Studies 44, no. 1: 45–55. 10.1111/j.1745-4603.2012.00364.x. 35484804

[jtxs70078-bib-0040] Smirnov, V. , D. Khramova , E. Chistiakova , et al. 2024. “Texture Perception and Chewing of Agar Gel by People With Different Sensitivity to Hardness.” Gels 11, no. 1: 5. 10.3390/gels11010005. 39851976 PMC11764599

[jtxs70078-bib-0041] Stokes, J. R. , M. W. Boehm , and S. K. Baier . 2013. “Oral Processing, Texture and Mouthfeel: From Rheology to Tribology and Beyond.” Current Opinion in Colloid and Interface Science 18, no. 4: 349–359. 10.1016/j.cocis.2013.04.010.

[jtxs70078-bib-0042] Sura, L. , A. Madhavan , G. Carnaby , and M. A. Crary . 2012. “Dysphagia in the Elderly: Management and Nutritional Considerations.” Clinical Interventions in Aging 7: 287–298. 10.2147/CIA.S23404. 22956864 PMC3426263

[jtxs70078-bib-0043] Szczesniak, A. S. 2002. “Texture Is a Sensory Property.” Food Quality and Preference 13, no. 4: 215–225. 10.1016/S0950-3293(01)00039-8.

[jtxs70078-bib-0044] Tanimoto, Y. , M. Watanabe , M. Saitou , et al. 2007. “Muscle Mass as an Indicator of Health Status of Community‐Dwelling Elderly Persons in Japan.” Bulletin of the Osaka Medical College 53, no. 2: 115–121. 10.57371/00000381.

[jtxs70078-bib-0045] Tonni, I. , G. Riccardi , M. G. Piancino , C. Stretti , F. Costantinides , and C. Paganelli . 2020. “The Influence of Food Hardness on the Physiological Parameters of Mastication: A Systematic Review.” Archives of Oral Biology 120: 104903. 10.1016/j.archoralbio.2020.104903. 33142153

[jtxs70078-bib-0046] van der Bilt, A. 2011. “Assessment of Mastication With Implications for Oral Rehabilitation: A Review.” Journal of Oral Rehabilitation 38, no. 10: 754–780. 10.1111/j.1365-2842.2010.02197.x. 21241351

[jtxs70078-bib-0047] van der Bilt, A. , J. H. Abbink , F. Mowlana , and M. R. Heath . 1993. “A Comparison Between Data Analysis Methods Concerning Particle Size Distributions Obtained by Mastication in Man.” Archives of Oral Biology 38, no. 2: 163–167. 10.1016/0003-9969(93)90202-w. 8476346

[jtxs70078-bib-0048] Yokohama, Y. , M. Sasaki , K. Kamata , Y. Takahashi , and Y. Tamada . 2024. “Method for Evaluating Muscle Fatigue From Multi‐Channel Surface Electromyography Signals of Suprahyoid and Infrahyoid Muscles During Swallowing.” Advanced Biomedical Engineering 13: 152–162. 10.14326/abe.13.152.

